# Diagnosis and treatment of Watershed strokes: a narrative review

**DOI:** 10.25122/jml-2023-0127

**Published:** 2023-06

**Authors:** Oana Andreea Dogariu, Ioan Dogariu, Corina Maria Vasile, Mihaela Corina Berceanu, Victor Cornel Raicea, Carmen Valeria Albu, Ioana Andreea Gheonea

**Affiliations:** 1 University of Medicine and Pharmacy, Craiova, Romania; 2 Department of Neurology, Emergency County Hospital, Targu-Jiu, Romania; 3 Department of Pediatric and Adult Congenital Cardiology, University of Bordeaux, Bordeaux, France; 4 Department of Cardiology, County Hospital, Craiova, Romania; 5 Department of Neurology, Neuropsychiatry Hospital, Craiova, Romania; 6 Department of Radiology, Emergency County Hospital, Craiova, Romania

**Keywords:** ischemic stroke, carotid stenosis, hypoperfusion, Watershed infarcts, ACA - anterior cerebral artery, BMT - best medical treatment, CAS - carotid artery stentings, CBF - cerebral blood flow, CBV - cerebral blood volume, CBZ - cortical border-zone, CEA - carotid endarterectomy, CT - computer tomography, DWI - diffusion-weighted imaging, ECST - European Carotid Surgery Trial, FLAIR - fluid attenuation inversion recovery, IBZ - internal border-zone, ICA - internal carotid artery, LVO - large vessel oclussion, MCA - middle cerebral artery, MS - multiple sclerosis, NIHSS - National Institute of Health Stroke Scale, MRI - magnetic resonance imaging, PCA - posterior cerebral artery, PET - positron emission tomography, SPECT - single photon emission tomography, TIA - transient ischemic attack, TOF - time of flight, WI - Watershed infarcts

## Abstract

Watershed strokes have been described previously as ischemic strokes located in vulnerable border zones between brain tissue supplied by the anterior, posterior, and middle cerebral arteries in the distal junction between two non-anastomotic arterial territories. Ischemic strokes in border zones are well-recognized entities and well-described in terms of imaging features, but the pathophysiological mechanism of brain injury production is not fully defined. Border zone ischemia is caused by cerebral hypoperfusion through decreased cerebral blood flow and arterial embolism in unstable atheroma plaque. It is often difficult to say which mechanisms are fully responsible for producing cerebral ischemic lesions. This review aimed to highlight the imaging aspect of watershed strokes and to correlate the clinical characteristics of this type of stroke with the diagnostic algorithm for optimal therapeutic management. Neurologists should promptly recognize this type of stroke and investigate its etiology in the shortest possible time.

## INTRODUCTION

Cerebral watershed (or border zone) infarcts (WI) were first discussed in 1883 [1] and typically involve the junction between the distal fields of two non-anastomosing arterial systems. These areas represent hemodynamic risk zones, where perfusion pressure is at its lowest. There are two types of WI: cortical WI, occurring at the junction of cortical territories supplied by the anterior, middle, and posterior cerebral arteries, and internal WI, occurring in the white matter between the deep and superficial territories of the middle cerebral artery [2]. Although rarely fatal, border zone infarcts have been observed in 19% to 64% of imaging studies focusing on severe internal carotid artery (ICA) disease [3], making them more prevalent than generally diagnosed in clinical practice. The symptoms of watershed strokes can have a progressive course or fluctuate. Sometimes the clinical picture is mild and can be overlooked by the clinician. The appearance of early motor focal seizures is a characteristic feature of cortical border zone infarcts [4]. The pathogenesis is not fully elucidated; the embolic or hemodynamic mechanisms are postulated [5]. Due to their subacute onset and symptoms that can evolve gradually over several hours or days, along with the association with focal clonic seizures or syncope [2], diagnosis of WI can be challenging for clinicians. However, WI accounts for up to 10% of all cerebral infarcts in autopsy studies [6], underscoring the importance of recognizing these infarcts to ensure adequate treatment in the acute phase. During this phase, diffusion-weighted imaging (DWI) MRI allows a more precise diagnosis [7].

## MATERIAL AND METHODS

This review aimed to outline the importance of properly recognizing Watershed stroke, an entity often overlooked in clinical practice and diagnostic considerations. A systematic search was conducted in the PubMed database to identify relevant articles using the keywords “watershed strokes” and “border zone infarcts” along with “carotid artery stenosis” and “artery-to-artery” embolism. No restrictions were placed on the publication date, study design, location, or inclusion/exclusion criteria. Only articles written in English and published before April 1, 2023, were included for analysis. Additionally, the reference lists of identified studies were reviewed, and the authors supplemented the search by including additional references based on their expertise in the field. Radiological images used in this review were sourced from the database of the Neurology Department at Emergency County Hospital Targu-Jiu.

### Anatomical vascularization of the cerebral territories

The intracranial circulation can be divided into an anterior portion and a posterior portion based on the two internal carotid and vertebral arteries, one on each side. The internal carotid artery (ICA) originates from the common carotid artery, which bifurcates into the internal and external carotid arteries at the level of the thyroid cartilage and the C4 vertebra. The ICA enters the skull through the carotid canal, located in the petrous portion of the temporal bone. Branches of the ICA include the ophthalmic artery, the posterior communicating artery, the anterior choroidal artery, and the two terminal branches, respectively, the anterior cerebral artery and the middle cerebral artery [8].

**Figure 1:**
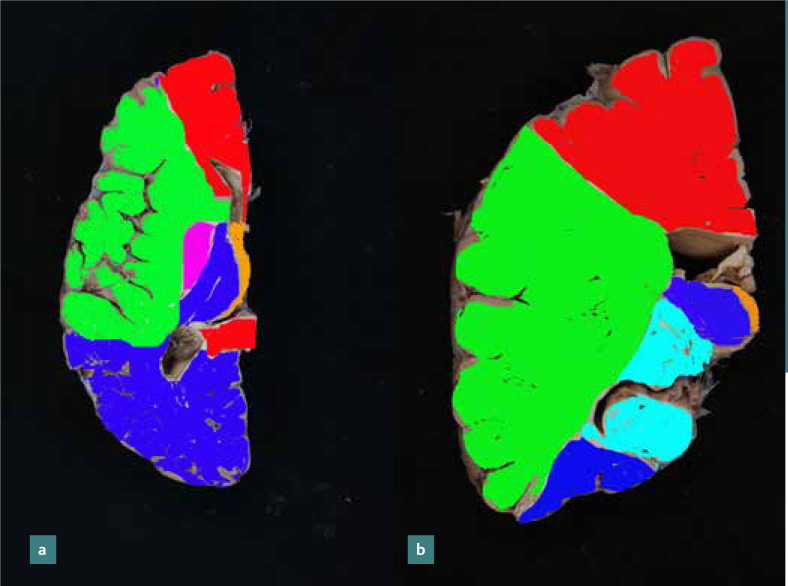
Arterial blood distribution: a - axial section at the level of the genu of the corpus callosum; b - coronal section at the level of the head of the caudate nucleus (anterior cerebral artery in red, the middle cerebral artery in green, the posterior cerebral artery in blue, the anterior choroidal artery in purple in the image a and light blue in image b, posterior communicating artery in orange). Regions between arterial territories (blue arrows) are prone to ischemia.

The anterior cerebral artery supplies the medial portion of the frontal and parietal lobe and the anterior portion of the corpus callosum, basal ganglia, and internal capsule. The middle cerebral artery, through its cortical branches, irrigates the lateral surface of the cerebral hemispheres except for the portion supplied by the anterior cerebral artery and the lower portion of the temporal lobe, which receives blood from the posterior cerebral artery. The middle cerebral artery also supplies the upper part of the head and body of the caudate nucleus, most of the globus pallidus, and putamen through its deep branches, known as lenticulostriate arteries. In addition, it supplies blood for the anterior arm of the internal capsule and partially for the posterior arm of the capsule (together with branches from the anterior choroidal artery) (Figure 1 a-b).

The vertebral arteries supply the medulla oblongata, pons, and cerebellum and then join at the level of the bulbo-pontine sulcus to form the basilar trunk. The posterior cerebral artery is the terminal branch of the basilar artery, responsible for vascularizing the occipital lobe and the posteromedial portion of the temporal lobe.

Border zone strokes depend on vascular anatomy, and variations in vascularization can lead to different stroke locations. A hypoplastic anterior or posterior communicating artery can lead to an incomplete circle of Willis that cannot compensate for proximal vessel occlusion. Variations of vessel origin give rise to atypical stroke syndromes. Patients may have posterior circulation syndromes because of ICA pathology when the posterior cerebral artery (PCA) arises from the ICA (so-called fetal PCA). In patients with a hypoplastic or aplastic A1 segment of the anterior cerebral artery (ACA), unilateral carotid or ACA disease can lead to bilateral frontal infarcts, which can also occur when the A1 segments for each side fuse to form a single A2 segment (an azygous ACA). Variations in the number of vessels can lead to clinical-imaging discrepancies that should prompt the search for these variations. Trifurcations of the ACA and the middle cerebral artery (MCA) are more common and should be considered in patients with apparently normal bifurcation but an incomplete filling of the vascular territory on computed tomography angiography (CTA) or digital subtraction angiography (DSA) [9].

## ANATOMY OF BORDER ZONE INFARCTS

### External Watershed Strokes

**Figure 2:**
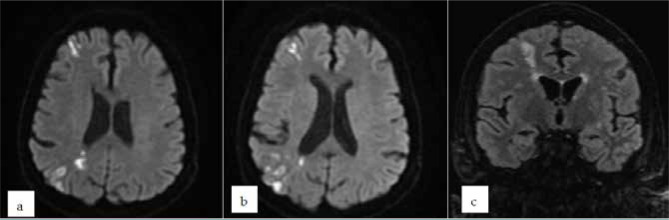
Brain MRI of a patient with external cortical border zone strokes in the watershed territories between right MCA/ACA and MCA/PCA on a symptomatic ICA stenosis (a, b - DWI axial sections, c - FLAIR coronal section)

External or cortical border zone strokes (CBZ) are due to microemboli, sometimes associated with cerebral hypoperfusion [10]. They are localized between the ACA/MCA and MCA/PCA distribution territories [11]. These infarcts have a triangular, cortical or cortico-subcortical, periventricular shape (Figure 2 a-c). Histopathologically, laminar cortical necrosis occurs as necrosis of neurons in a situation where oxygen and glucose requirements are inadequate, as in cardiac arrest, global hypoxia, or hypoglycemia. Topographically, the infarcts between the ACA and MCA border territories are located in the frontal cortex, extending from the frontal horn of the lateral ventricle to the cortex. The infarcts between the MCA and the PCA border territories are distributed in the parieto-occipital region, extending from the occipital horn of the lateral ventricle to the cortex. In cases of diffuse cerebral hypoperfusion, infarcts can also occur in the subcortical white matter, particularly at the level of parallel parafalciform areas. Regarding prognosis, this type of stroke has a less noisy clinical course and a favorable outcome.

The external cortical infarcts show a bimodal spatial distribution. Anteriorly, they are focused on the posterior central lobe at the junction between the central sulcus and the precentral sulcus. The posterior cortical border infarcts are centered at the level of the superior parietal lobe, postero-lateral to the postcentral sulcus, and the prevalence of border zone infarcts is low between these two areas. Junctional strokes spare the medial cortex.

### Internal (Deep) Watershed Strokes

Internal border zone strokes (IBZ) primarily occur due to hypoperfusion and are located between the distribution areas of the three main arteries: ACA, MCA or PCA, and the perforating, lenticulostriate arteries, Heubner's recurrent artery, and the branches of the anterior choroidal artery. Half of the deep watershed strokes and MCA occlusion can have an anatomical variation of a common stem origin of lenticulostriate arteries [12].

**Figure 3:**
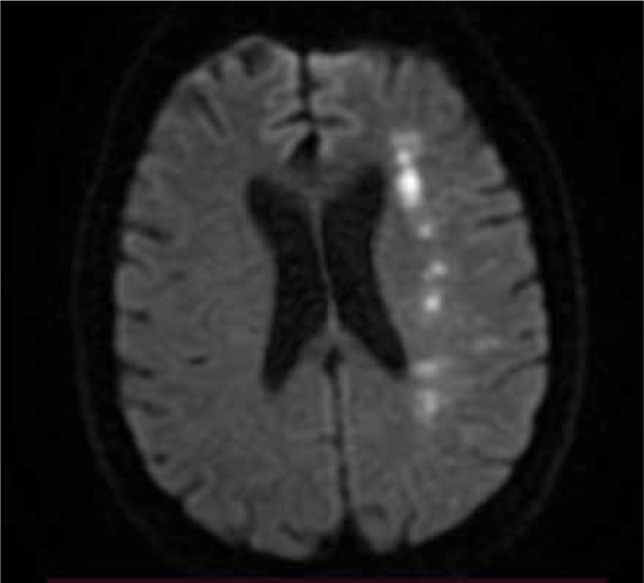
Brain MRI, axial DWI – Patient with confluent deep watershed ischemic stroke

Generally, more than three lesions should be present, each with a diameter of at least 3 mm. These lesions are distributed linearly, parallel to the lateral ventricle, located at the level of the semioval center or corona radiata. Multiple white matter strokes arranged parallel to the lateral ventricle are associated with severe hemodynamic impairment and are likely due to hemodynamic mechanisms [13]. Occasionally, the lesions can become confluent, creating a band imaging appearance, the "string of pearls" pattern pathognomonic. The "string of pearls" sign is visualized on DWI, and T2/FLAIR sequences represent a type of border zone stroke between cortical penetrating arteries and ascending perforating arteries [14] (Figure 3). Depending on the imaging appearance, deep territorial border infarcts may be confluent or partial [15]. Partial infarcts tend to adopt a “rosary-like” pattern on brain imaging, which can sometimes be difficult to differentiate from striato-capsular infarcts, lacunar infarcts, or leukoaraiosis. In terms of outcome, they are linked to higher morbidity and an increased risk of recurrent ischemic stroke. There may be some differences between the pathogenesis of confluent internal WS, and partial internal WS. Microembolism may contribute to confluent watershed infarcts and hypoperfusion, which is the main pathogenesis of partial infarcts [16].

### Cerebellar Watershed Strokes

Watershed infarcts within the cerebellum are usually smaller than 2 cm. They are typically found in the marginal areas of the anterior inferior cerebellar artery, superior cerebellar artery, posterior inferior cerebellar artery, and their branches. These infarcts are usually caused by stenosis or embolism, and embolic events may be cardiac or due to atherosclerotic disease or dissection of an artery in the vertebrobasilar system [17]. Cerebellar watershed infarcts can rarely be observed in clinical practice, and their incidence has not been established.

## PATHOPHYSIOLOGY

While the anatomical, pathological, and imaging aspects of border zone infarcts are well-known and studied, their pathogenesis remains a matter of debate. This type of stroke can be triggered by circumstances that induce hypotension or hypovolemia. The mechanism of border zone ischemia is cerebral hypoperfusion through decreased cerebral blood flow and arterial embolism [18]. In an unstable atheroma, plaque in the internal carotid artery usually causes significant stenosis. Border zone ischemic strokes due to cerebral hypoperfusion can occur through a global decrease in cerebral blood flow in a systemic context, for example, following resuscitated cardiac arrest or prolonged arterial hypotension, in which cases ischemic lesions tend to be bilateral in brain tissue. Triggers for hypotension are often precipitating factors, such as rising from a lying position, especially in patients with dysautonomia (e.g., diabetic patients, patients with Parkinson’s [19] disease or Parkinson's plus syndrome - especially multiple system atrophy), Valsalva maneuvers, physical exertion, administration of antihypertensive drugs or dopamine agonists or L-Dopa, anemia, heavy bleeding, and severe dehydration. In tight carotid stenosis, blood flow will be insufficient to irrigate the vulnerable border areas between the brain's main arteries. There is also the possibility of ischemic lesions in an occluded carotid artery (internal or common) with a patent polygon of Willis, which sometimes becomes nonfunctional and collateral circulation is ineffective. Repeated episodes of systemic hypotension in a patient with atheromatous or carotid stenotic carotid occlusion [20], usually elderly, represent the typical scenario of ischemic stroke in watershed areas, with the predilection site centered on deep border areas. A study showed that a history of cerebral infarction, high levels of low-density lipoprotein cholesterol, and ophthalmic artery reflux (on transcranial Doppler) were independent risk factors for internal border zone infarcts in patients with symptomatic chronic internal carotid artery occlusion [21]. Carotid artery dissection leading to ICA stenosis can rarely be incriminated as a mechanism of producing border zone infarction. Several cases of fibromuscular dysplasia leading to border zone infarcts are also reported in the literature [22].

Seok Woo Yong *et al*. performed a study on 946 patients with ischemic stroke in the distribution territory of the middle cerebral artery [23]. They identified 45 patients with border zone ischemic strokes in the superficial, cortical territory and 45 ischemic strokes in the internal watershed deep territory. They found a stronger association between deep junctional infarcts and stenosis or occlusion of the internal carotid artery or the middle cerebral artery. Clinical deterioration in the first seven days and worse prognosis at three months were observed more frequently in patients with infarcts in the deep border zones than those with infarcts in the superficial border zones. They compared the data obtained with data from 4 other relevant studies that compared the pathophysiological differences between deep and superficial watershed infarctions. They concluded that internal watershed strokes are most often caused by hemodynamic compromise, while external ones most often have an embolic cause.

## CLINICAL ASPECTS

Symptoms of a watershed stroke can include weakness or paralysis of one leg and arm on the same side of the body, vision loss in half of the field of vision, slurred speech, facial drooping, and conjugate eye deviation.

Sometimes at the onset of a stroke, the patient may experience syncope or near syncope, which supports the theory of hemodynamic failure. Watershed strokes are often encountered in the elderly because of hemodynamic disturbances like postural hypotension, cardiac arrhythmias, and excessive use of antihypertensive drugs. The typical clinical presentation is a progressive, fluctuating, or episodic decrease in muscle strength in the hand or upper limb, occasionally associated with focal seizures. It is cited in the literature that seizures are more frequent in watershed strokes than in other types of strokes.

Ischemia on the border between the MCA/PCA territories can produce infarcts in the parietal-temporal areas, clinically manifesting as cortical blindness or fragments of Balint syndrome (oculomotor apraxia, optic ataxia, and simultaneous agnosia) or Gerstmann syndrome (agraphia, acalculia, digital agnosia, and left/right disorientation). Sensory transcortical aphasia can also occur (impaired understanding with the preservation of fluency and repetition). Persons with chronic multiple microinfarcts in cortical watershed regions have a lower cognitive function in working memory and visuospatial abilities [24]. While subcortical and capsule-thalamic watershed strokes can mimic a lacunar syndrome due to small-vessel disease, cerebellar border zones infarctions are associated with non-specific vertigo syndrome or ataxia, and in brainstem infarction patients are comatose with other signs of the brainstem [25].

Bilateral border zone infarctions between the ACA/MCA territories can produce proximal muscle weakness in the upper limb (the characteristic "man in the barrel" syndrome) [26], which is caused by bilateral symmetric damage isolated to the upper extremity motor fibers in the motor cortex, corona radiata, internal capsule, and basal ganglia. The topographical differential diagnosis of this syndrome is made with lesions at the cervical cord level, with bilateral lesions of the brachial plexus or peripheral neuropathies.

## STAGES OF HEMODYNAMIC IMPAIRMENT

Two stages of hemodynamic impairment are described regarding the magnitude of the decrease in cerebral perfusion pressure. In stage I, a reduction of cerebral perfusion pressure will lead to compensatory vasodilatation of the resistance cerebral vessels in the brain. Doppler ultrasonography, perfusion CT, MRI, SPECT, or PET can measure this physiological response to decreased perfusion pressure. These methods can measure cerebral blood flow (CBF), cerebral blood volume (CBV), and mean transit time. The increase in CBV and the increase in the mean transit time are characteristics of stage I.

In stage II, the additional reduction of cerebral perfusion pressure causes impaired compensatory vasodilatation, which causes cerebral blood flow to decrease. As blood flow decreases, the extraction oxygen fraction in the cerebral parenchyma will increase. PET can measure this growth. Calculating the fraction of extracted oxygen provides information about the patient's hemodynamic status with cerebrovascular disease. The increased extracted oxygen fraction in a brain region with ischemia is described as "misery perfusion". This phenomenon, in which there is a reduction in blood flow and an increase in the extracted oxygen fraction, is specific to stage II [27].

## IMAGING DIAGNOSIS

Non-contrast head CT is the first choice in the diagnostic algorithm for acute stroke in most centers due to its wide availability and speed of investigation. The main drawback is the low sensitivity in hyperacute phases. Early signs of ischemia in the hyperacute phase: parenchymal hypoattenuation, defined as a region of low density compared to similar structures in the contralateral hemisphere, e.g., loss of grey/white matter differentiation or basal ganglia hypoattenuation [28]. Changes in lentiform nuclei are visible in 75% of patients within 3 hours of onset [29]. At the cortical level, the insular area is the most sensitive and vulnerable to early ischemia (the insular ribbon is the characteristic appearance). Visualization of the loss of differentiation between gray matter and white matter is facilitated using a "stroke window" that has a narrow width and a slightly lower center than a routine brain window (width = 8, center = 32 H.U.) [28]. In the acute phase, parenchymal hypoattenuation and cerebral edema become evident. In the subacute phase (2-3 weeks), in 50% of patients, a "fogging" effect may occur [30], whereby ischaemic brain parenchymal density tends to recover (by migration of lipid- and leukocyte-rich macrophages, capillary proliferation and red blood cell extravasation).

CT angiography is a non-invasive method that enables imaging of the internal and external carotid vessels and vertebral arteries. Its main advantages are that it provides a better assessment of the morphology and characteristics of atheroma plaques. It is a more cost-effective investigation than conventional angiography (DSA), with lower risks of periprocedural complications. It also allows imaging of non-vascular structures at the cervical level and brain parenchyma. The disadvantages are that it has a lower resolution than conventional catheter angiography, which makes it more difficult to identify wall changes that occur in arterial dissections or vasculitis. Compared to magnetic resonance angiography, the disadvantage is that it is irradiating and requires a contrast agent. CTA can accurately calculate the degree of internal carotid artery stenosis [31].

**Figure 4:**
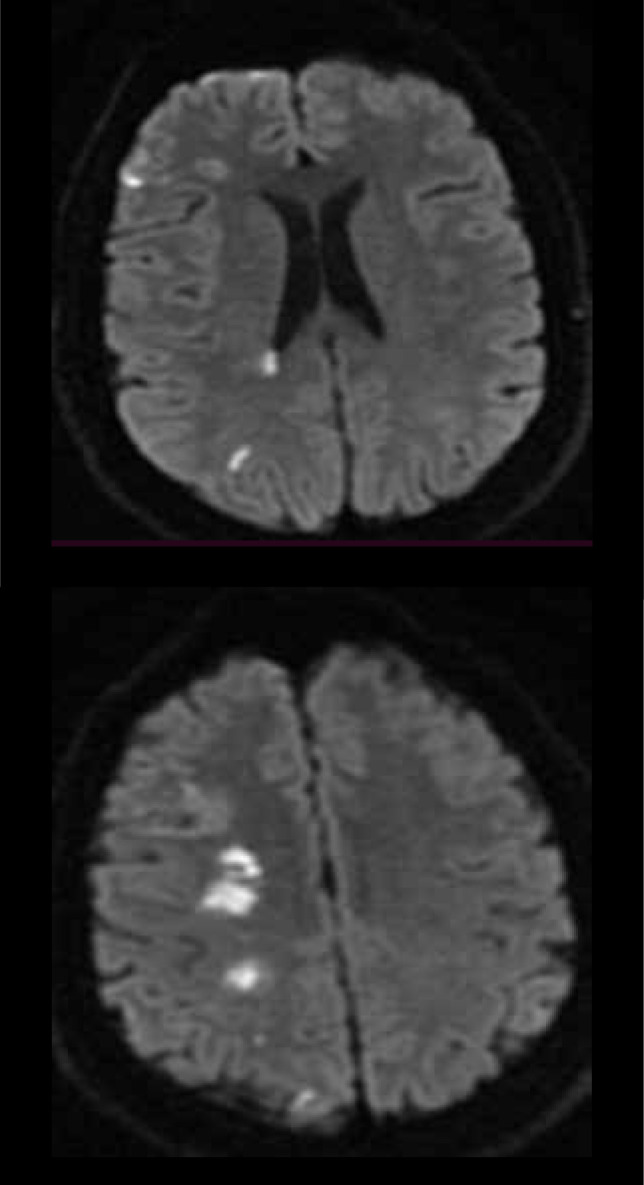
Brain MRI of a patient with acute ischemic stroke in the deep and superficial watershed territories of right ICA (DWI, axial sections).

**Figure 5:**
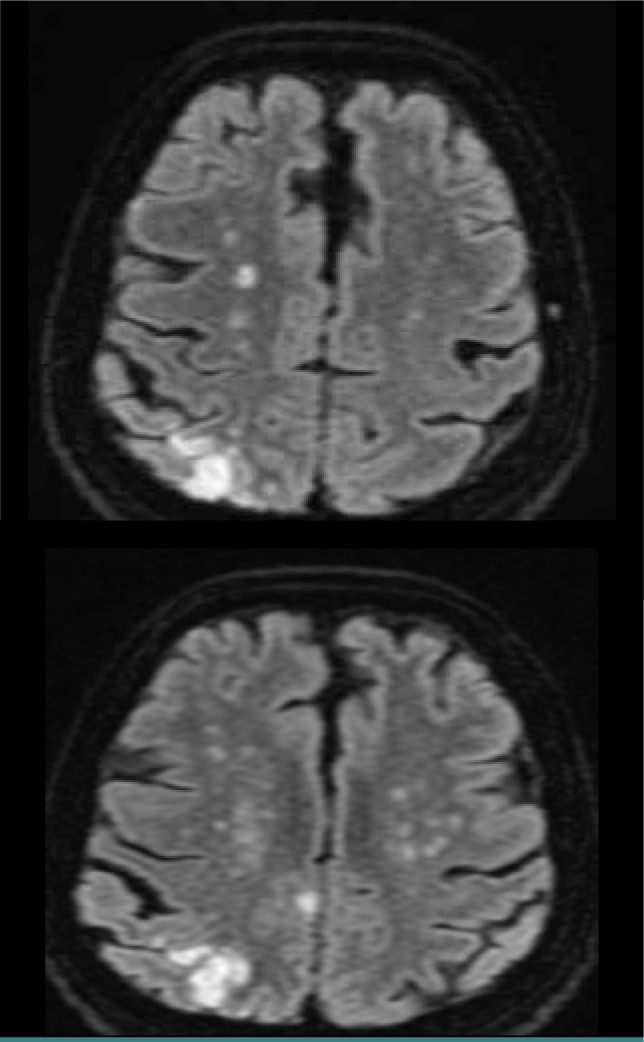
Brain MRI of a patient with internal (deep) border zone strokes (FLAIR axial sections). We can see confluent lesions in a band-like distribution parallel with the lateral ventricle (characteristic "string of pearls" sign). The lesions are more than 3 mm long, suggesting deep watershed stroke lesions.

Brain MRI has higher specificity and sensitivity than CT in detecting acute ischemic stroke, but it is a more time-consuming examination and is currently less available. In the early hyperacute phase, within minutes, DWI shows an increase in signal in the affected area that correlates well with the infarct core (Figure 4). In the late hyperacute phase, the signal will increase after 6 hours on T2 sequences, initially more easily seen on FLAIR (Figure 5). T1 hypointensity is noticed after 16 hours [32].

**Figure 6:**
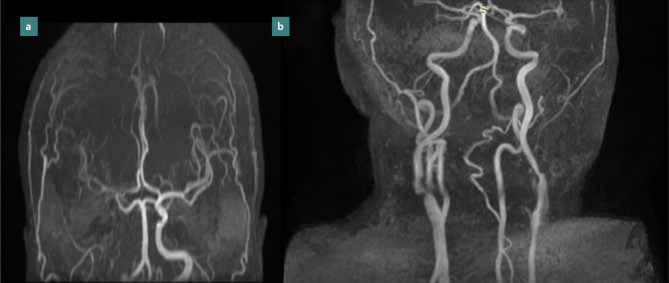
MRI 3D TOF with signal loss of the ICA in the petrous segment (a); 3D TOF with severe right internal carotid artery stenosis at the bifurcation (b).

MRI angiography can be performed either with contrast substance (contrast-enhanced MRI angiography-MRA) by administering gadolinium or without contrast substance (time of flight angiography 2D or 3D TOF). 3D TOF (Figure 6) imaging is the most used technique in cerebrovascular pathology. 3D TOF MRA gives pertinent information about the pathophysiology of internal watershed strokes, which can help in case management decisions [33]. 3D TOF MRA offers several advantages, including a relatively short acquisition time (2-5 minutes) and increased spatial resolution. However, it also has limitations to consider. These include the increased sensitivity to tissues with short T1 that can mimic flow, reduced sensitivity in visualizing vessels with slow flow (such as distal arteries), decreased sensitivity in capturing flow at the edges of the acquisition volume, and increased susceptibility to the saturation phenomenon of spines [34]. It should also be noted that there are some limits and disadvantages of MRA: limited visualization of small-caliber or tortuous vessels, overestimation of the degree of medium/tight stenoses, longer acquisition time than conventional angiography, with susceptibility to patient movement artifacts, and susceptibility to various artifacts: metal, air, proteinaceous liquid, bleeding.

**Figure 7:**
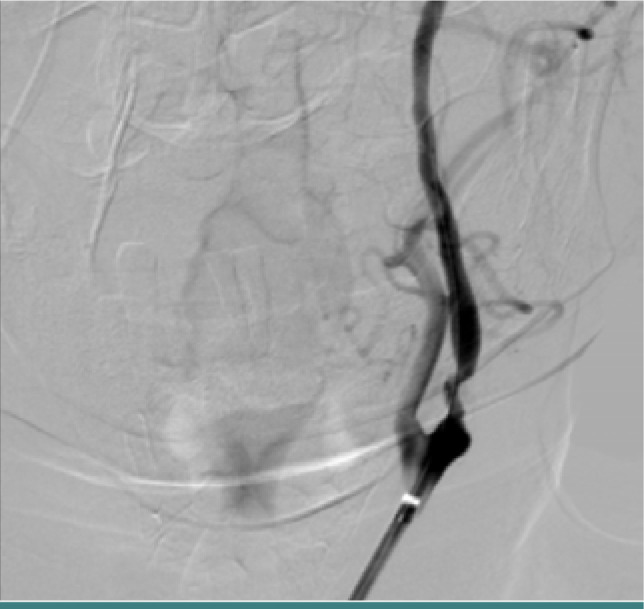
Digital subtraction angiography of a patient with severe left ICA stenosis in the proximal portion of the artery

Conventional angiography (digital subtraction angiography) is still considered the "gold standard" in cerebral vascular imaging. However, this invasive diagnostic method is associated with complications (1% incidence of neurological deficits). While currently less used for atherosclerotic carotid disease to confirm symptomatic carotid stenosis detected by non-invasive methods or to highlight hemodynamic changes caused by severe stenosis, DSA is essential for carotid artery interventions, especially for stenting at this level. Neuro-interventional endovascular procedures with DSA include mechanical thrombectomy in acute ischemic stroke, coiling of aneurysms, embolization of arteriovenous fistulae or malformations, and intra- or extracranial stenting [35] (Figure 7).

**Figure 8:**
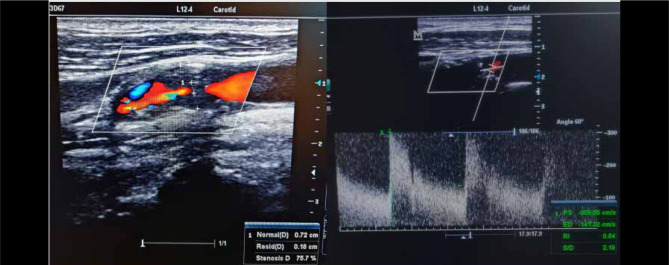
Doppler ultrasound of a patient with high-grade left ICA stenosis (>70% reduced diameter). The peak systolic and end-diastolic velocities are increased. The patient had an episode of amaurosis fugax and then developed hemiparesis due to a lesion in the border zone between MCA/ACA

Doppler ultrasound of the cervical vessels is the key investigation for screening carotid stenosis, assessing the macroscopic appearance of the plaques and their hemodynamic impact. This may highlight an atheroma plaque that may cause carotid stenosis or occlusion that may be responsible for border zone infarctions (Figure 8). The European method for quantification of stenosis grade (ECST) is a technique in which the vessel walls and initial anatomical boundaries are easy to highlight [36]. Hemodynamically significant carotid stenoses are further assessed by CT angiography or DSA for accurate quantification of the degree of stenosis.

## DIFFERENTIAL DIAGNOSIS

The main differential diagnosis of border zone infarcts is made with lacunar infarcts [37]. Lacunar ischemic strokes are small infarcts (<15 mm) in the distal distribution territories of deep penetrating arteries (lenticulostriates, thalamoperforates, pontine perforators). They are usually randomly distributed in the basal ganglia, thalamus, and pons, without having the characteristic localization of border zone infarcts. Multiple embolic infarcts can also have a similar imaging appearance with strokes in the border zone territories, but they are usually multi-territorial and bilateral. Small vessel disease, as seen in CADASIL, CARASIL, MELAS, or Fabry disease, is part of the differential diagnosis of watershed strokes. Demyelinating diseases like multiple sclerosis (MS) can cause symptoms similar to those seen in border zone strokes, like hemiparesis, dysarthria, vertigo, or ataxia. MRI helps distinguish the presence of plaques due to MS from a stroke. The lesions in MS tend to be located cortical/ juxtacortical and periventricular along the axis of the medullary vein perpendicular to the body of the lateral ventricles.

## MANAGEMENT

According to guidelines, in the acute phase of an ischemic stroke, supposed to be in a watershed territory, intravenous thrombolysis with alteplase is recommended in the first 4.5 hours from the onset of symptoms if the patient is eligible. Patients with acute ischemic stroke due to large vessel occlusion (LVO) in the anterior circulation with symptoms onset in the last 6 hours should be considered for mechanical thrombectomy with stent retriever or direct aspiration. In patients with acute ischemic stroke caused by LVO with symptoms onset between 6 and 16 hours, mechanical thrombectomy is recommended if they meet the eligibility criteria of DAWN and DEFUSE 3. Also, in patients with acute ischemic stroke in the last 16-24 hours who meet DAWN criteria, it is reasonable to consider mechanical thrombectomy.

In cases of watershed infarctions within the past 6 months caused by carotid artery stenosis ranging from 70% to 99%, the American Heart Association (AHA) guideline [38] provides a class I, level A recommendation for endarterectomy, provided that the risk of peri-operative morbidity and mortality remains below 6% [38]. Similarly, for carotid stenosis between 50% and 69% presumed to be implicated in stroke etiology based on catheter-based or non-invasive imaging, endarterectomy is considered (class I, level B). As an alternative to carotid endarterectomy, carotid stenting should be considered depending on anatomical features or associated medical conditions (radiotherapy-induced carotid stenosis or restenosis after endarterectomy). In case of carotid stenosis below 50%, endarterectomy or stenting is not recommended. While endarterectomy has a modest effect on asymptomatic carotid stenosis, the role of stenting is uncertain and remains to be further established in these cases [39]. Patients with carotid stenosis and stroke should take intensive medical treatment with antiplatelet agents, lipid-lowering agents, hypertension treatment, and control of diabetes [40]. Reiff T. *et al*. [41] published a paper in 2022 comparing carotid endarterectomy (CEA) plus best medical treatment (BMT) versus carotid stenting (CAS) plus BMT versus BMT alone in terms of 30-day prognosis in patients with asymptomatic carotid artery stenosis of ≥70% European Carotid Surgery trial criteria. 513 patients were recruited, of which 203 underwent CEA + BMT, 197 CAS + BMT and 113 BMT. It was concluded that CEA + BMT and CAS + BMT are not superior to BMT alone regarding the risk of stroke or death at 30 days.

If cervical arterial dissection is responsible for an ischemic stroke in the border zone territories, according to the guidelines, either antiplatelet treatment (class I indication, level C) or oral anticoagulant treatment with warfarin (class 2a indication, level B) is recommended. Endovascular treatment is recommended in patients with arterial dissections who present recurrence of events despite antiplatelet therapy (class 2b, level C) [42].

In the case of a watershed ischemic stroke with an NIHSS score less than or equal to 3 or a TIA with an ABCD2 greater than or equal to 4, dual antiplatelet therapy with aspirin and clopidogrel is recommended for 30 to 90 days after the event, ideally initiated in the first 12-24 hours, up to 7 days after the start of the event.

In selected patients, in the case of a moderate ischemic stroke (NIHSS less than or equal to 5) or a transient ischemic attack with an ABCD2 score greater than or equal to 6 or symptomatic intracranial or extracranial ≥30% stenosis of an artery that could account for the event the same guideline recommends dual antiplatelet treatment [43] with aspirin + ticagrelor started in the first 24 hours and maintained for 30 days.

Anticoagulant treatment has no guideline indication for border zone strokes, even if an artery-to-artery embolic etiology is suspected.

**Figure 9:**
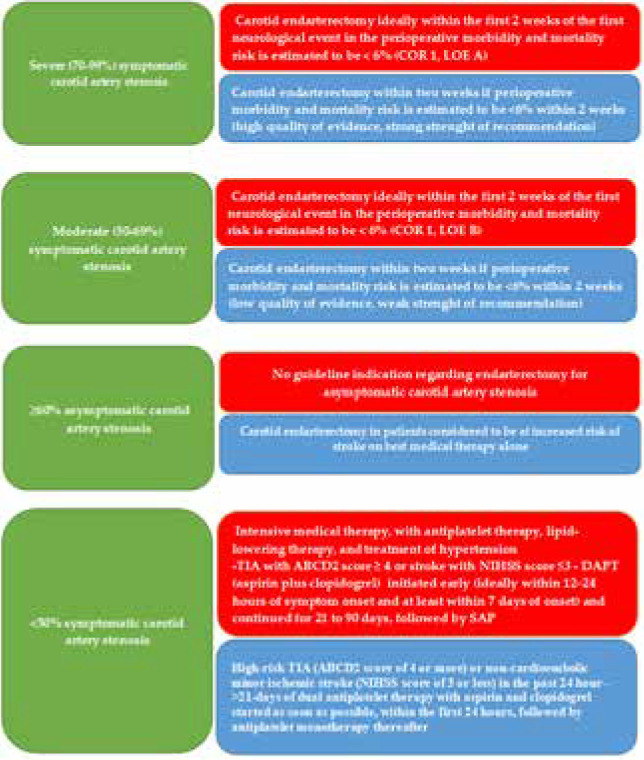
Comparative between AHA [38] (red) and ESO [44,45] (blue) guideline recommendations of management measurements in carotid stenosis, which can be responsible for watershed strokes.

We compared the AHA [38] and ESO [44, 45] guidelines on the management of symptomatic carotid artery stenosis (Figure 9).

## LIMITATIONS

However, it is important to acknowledge that the analysis conducted in this study has certain limitations. A deeper understanding of anatomical variations and the underlying pathophysiological mechanisms of watershed strokes is crucial for enabling faster diagnosis and more effective therapeutic interventions. Current research is limited because the location of watershed strokes may sometimes be difficult to define. This difficulty arises from the fact that vascular territories can vary between individuals, making it challenging to establish consistent anatomical patterns of vascularization. We have not investigated clinical symptoms concerning anatomical patterns of vascularization.

## CONCLUSION

Neuroimaging remains essential in stroke diagnosis and provides rapid etiology and therapeutic management guidance. Ischemic strokes in borderline territories are a well-known and described imaging entity, but the pathophysiological mechanism of brain injury is not fully defined. Artery-to-artery embolism downstream of an unstable atheroma plaque and tissue hypoperfusion with stenosis and hypotensive arterial overload is incriminated as production mechanisms. It is often difficult to say which mechanisms are fully responsible for generating cerebral ischemic lesions, the two coexisting and acting synergistically.

Anatomically, there are two types of watershed infarcts: external (cortical) and internal in the deep white matter. The external ones have an ex-triangular shape tending from the frontal or occipital horn of the lateral ventricle towards the cortex. Internal border zone infarcts may be confluent or partial. Confluent infarcts form a linear band along the lateral ventricles ('string of pearls' appearance), while partial infarcts are more discrete ('rosary lesions').

Advanced imaging techniques, such as diffusion and perfusion MRI, PET, perfusion CT, and transcranial Doppler ultrasonography, assist in understanding the pathophysiology of these infarcts and the degree of damage to cerebral hemodynamics, thereby guiding management.

Regarding management, we have clear guidelines on the best medical treatment and revascularization according to the degree of stenosis. However, guidelines still do not specify the optimal blood pressure level in symptomatic carotid stenosis with a watershed stroke until endarterectomy. A better understanding of the pathophysiology of WS infarction could guide treatment.

## Personal thanks

This study is part of the doctoral thesis of Dr. Dogariu Oana Andreea from the University of Medicine and Pharmacy of Craiova, Craiova, Romania.
